# Distinct Stage Changes in Early-Life Colonization and Acquisition of the Gut Microbiota and Its Correlations With Volatile Fatty Acids in Goat Kids

**DOI:** 10.3389/fmicb.2020.584742

**Published:** 2020-10-09

**Authors:** Jiazhong Guo, Pengfei Li, Ke Zhang, Lin Zhang, Xiaolong Wang, Li Li, Hongping Zhang

**Affiliations:** ^1^College of Animal Science and Technology, Sichuan Agricultural University, Chengdu, China; ^2^College of Animal Science and Technology, Northwest A&F University, Xianyang, China

**Keywords:** fecal microbiota, establishment, acquisition, volatile fatty acid, rumen microbiota, early life, goat

## Abstract

In livestock, a comprehensive understanding of the early-life establishment and acquisition of commensal gut microbiota allow us to develop better husbandry management operations and manipulate the gut microbiota for young animals, improving the efficiency of animal production. Here, we collected 123 microbial samples of 11 healthy goat kids and their mothers to investigate the colonization and acquisition of the gut microbiota and their correlations with volatile fatty acids (VFAs) in goat kids from birth to day 56. An age-dependent increasing and more homogeneous diversity were observed for the feces of goat kids. Overall, Firmicutes, Bacteroidetes, and Proteobacteria were the predominant phyla in the fecal microbiota of goat kids, but their relative abundance varied considerably with age. Accordingly, the colonization of the fecal microbiota in goat kids was divided into three distinct stages: newborn (day 0), non-rumination stage (days 7–21), and transition stages (days 28–56). LEfSe analysis revealed a total of 49 bacterial biomarkers that are stage-specific (LDA score > 3, *P* < 0.05). Significant Spearman correlations (*P* < 0.05) were observed between the abundances of several bacterial biomarkers and the VFA concentrations. Furthermore, a substantial difference in the fecal microbiota composition was present between 56-day-old goat kids and mothers, whereas there was a moderate difference in the rumen microbiota between them. Among four body sites (i.e., feces, oral cavity, vagina, and breast milk) of mothers, the maternal vaginal and breast milk microbiota were the major source of the fecal microbiota of goat kids in the first 56 days after birth, although their contributions decreased with age and unknown sources increased after day 28. In summary, we concluded that the gut bacterial community in goat kids after birth was mainly acquired from the maternal vagina and breast milk. Its colonization showed three distinct phases with dramatic shifts of composition mainly driven by age and diet changes. Our results provide a framework for a better understanding of the roles of the gut microbiota in young ruminants.

## Introduction

The gastrointestinal tract of mammals is inhabited by trillions of microbes that play essential roles in host physiology, development, and health (Guarner and Malagelada, 2003; Lynch and Pedersen, 2016). In adult ruminants (e.g., cattle and goat), the foregut (i.e., rumen) and the hindgut (i.e., large intestine) both serve as major reservoirs for gut microbiota, which is substantially different from that in adult humans and monogastric mammals. The rumen microbiota plays primary roles in the digestion of fibrous feed that is required for adult ruminants (Russell and Hespell, 1981), whereas the intestinal microbiota is involved in the digestion and absorption of rumen bypass nutrients (e.g., bypass starch and protein) and the production of methane (Buddle et al., 2011).

The establishment and temporal dynamics of the healthy rumen microbiota have been well characterized in normal cattle (Li et al., 2012; Jami et al., 2013; Rey et al., 2014) and goats (Han et al., 2015; Jiao et al., 2015a,b; Wang et al., 2016; Zhang et al., 2019) during different growth stages. Particularly, the initial establishment of the gut microbial community in ruminant kids after birth generally occurs in three successive stages: the initial colonization stage, the transitional stage, and the mature stage (Buddle et al., 2011), according to the observations on the rumen microbiota in early life (Li et al., 2012; Jami et al., 2013; Rey et al., 2014; Jiao et al., 2015b; Zhang et al., 2019). However, the initial colonizers of the gut microbiota depend on the delivery mode (i.e., vaginal delivery or cesarean section), feeding-management operations, and the surrounding environments (Arrieta et al., 2014). For example, calves in direct contact with their mothers obtain microbes through the mouth, feces, skin, and milk that are not available for individuals raised in isolation (Yeoman et al., 2018). Late-weaning (i.e., 8 weeks of age) results in a more gradual shift in microbial diversity in both the rumen and feces of dairy calves, compared to early-weaning (i.e., 6 weeks of age) (Meale et al., 2017). A better understanding of the acquisition of microbes for newborn livestock is needed, which allows us to develop more advanced methods to manipulate the gut microbiota and would help improve the health and productivity in the later life of animals.

The intestinal microbiota is particularly significant for young ruminants in early life because ruminants are born without a functional rumen, and a symbiotic ruminal microbial community is not established until the first few months of life (Wardrop and Coombe, 1960; Lane et al., 2002; Huws et al., 2018). As an almost exclusive diet for preweaning ruminants, milk is only digested and absorbed in the intestine due to closure of the esophageal groove by reflex action (Huws et al., 2018). More importantly, the intestinal microbiota produces a variety of metabolites, especially for volatile fatty acids (VFAs) that can be used as energy sources and signaling molecules in hosts (den Besten et al., 2013; Koh et al., 2016). Furthermore, fecal microbiota transplantation can reduce bowel permeability and thereby relieve the severity of ulcerative colitis in humans (Shen et al., 2018), indicating an important relationship between the gut microbiota and gastrointestinal diseases. In the goat industry, diarrhea diseases often resulting from dysbiotic intestinal microbiota driven by increased pathogen infection are a major cause of high mortality rates for pre-weaning goat kids. Thus, the characterization of healthy gut microbiota in normal individuals would provide baseline data for a better understanding of gut diseases induced by microbial dysbiosis.

Although the colonization dynamics of the hindgut microbiota (Jiao et al., 2016; Li et al., 2019) have been explored in goats kids during the first 8 weeks after birth (i.e., from birth up to the rumination phase), the sample sizes or sampling time points were limited in these studies. Furthermore, the absence of the ruminal and fecal microbiota of adult ruminants in the same studies hampered the evaluation of the maturity levels of the gut microbiota. We still lack a comprehensive understanding of the early-life acquisition and establishment of the gut microbiota in goat kids. In the current study, we collected 123 microbial samples of 11 healthy goat kids and their mothers (*n* = 9) across multiple body sites to investigate the establishment and the acquisition of the gut microbiota and the temporal dynamics of VFAs in healthy goat kids during the first 8 weeks of age.

## Materials and Methods

### Ethics Statement

The experiments involving animals in this study were performed in agreement with the guidelines and regulations for the Administration of Affairs Concerning Experimental Animals (Ministry of Science and Technology, China). All experimental protocols were approved by the Institutional Animal Care and Use Committee of the College of Animal Science and Technology, Sichuan Agricultural University (No. DKYB20081003).

### Animals and Sample Collection

To systematically investigate the acquisition and development of the gut microbiota in goat kids from birth to 56 days old, we selected nine healthy and two-parity Chengdu Brown mother goats with a similar delivery date (March 7 to March 17, 2019) in different pens on the Chengdu Brown goat breeding farm in Dayi county. On parturition day, we collected rectal feces (*n* = 9) of each mother goat and swabs of three other body sites: the oral cavity (*n* = 9), the vagina (*n* = 9), and the breast milk (*n* = 9), following the protocols described in a previous study (Ferretti et al., 2018). Before the collection of microbial samples, sterile cotton swabs were soaked with phosphate buffer saline. Fecal samples were taken directly from the rectum using sterile cotton swabs. To collect microbial samples in the oral cavity, we swabbed the gingiva, teeth, and oral mucus of each goat, and the swab heads were put into sterile plastic tubes. We obtained microbial samples in the vagina by swabbing the vaginal orifice for ∼30 s and the swab heads were put into sterile plastic tubes. Before the collection of raw milk, we cleaned udders of each goat with sterile water. We then collected approximately 5 ml of goat milk after discarding the first few milliliters. All samples were snap-frozen in liquid nitrogen and stored at −80°C until DNA extraction.

The nine mother goats included in this study delivered vaginally, giving birth to 11 goat kids (9 males and 2 females), and the goat kids lived with their mothers during the whole trial period. Based on clinical observations and body weight measurements, all goat kids were thought to be healthy, normal individuals during the trial period. According to the management on the farm, the diet of goat kids before 18–22 days old was almost entirely breast milk, although they were fed with *ad libitum* solid food (corn silages and fresh grasses) throughout the trial period. Afterward, the solid food intake of the goat kids gradually increased with age. We obtained rectal feces of the kids on days 0 (*n* = 9, sampling within 12 h after birth), 7 (*n* = 11), 14 (*n* = 11), 21 (*n* = 11), 28 (*n* = 8), 42 (*n* = 10), and 56 (*n* = 11) after birth. As described in our previous work (Guo et al., 2020), we also collected ∼30 ml of rumen fluid of each mother goat (*n* = 5) and 56-day-old goat kid (*n* = 11) using a stomach tube attached to a vacuum pump before the morning feeding.

### Microbial DNA Extraction and Measurement of VFAs

Microbial DNA was extracted from each of all the microbial samples (*n* = 123) from goat kids and mothers using the OMEGA E.Z.N.A. Stool DNA Kit following the manufacturer’s protocol (Omega Bio-Tek Inc., Norcross, GA, United States). DNA was quantified with a NanoDrop 2000 spectrophotometer (Thermo Fisher Scientific, United States), and the integrity was checked by 1% agarose gel electrophoresis. Concentrations of volatile fatty acids (VFAs) were determined using an Agilent 7820A gas chromatograph (Agilent Technologies, Santa Clara, United States) following a standard procedure described in previous work (Li et al., 2014).

### High-Throughput Sequencing and Data Analysis

To analyze the bacterial communities, the V3–V4 region of the 16S rRNA gene was amplified using a primer pair (341F: 5′-CCTAYGGGRBGCASCAG-3′ and 806R: 5′-GGA CTACNNGGGTATCTAAT-3′) (Sun et al., 2019), and the thermocycling protocol of the amplification was: 98°C for 1 min, followed by 30 cycles at 98°C for 10 s, 50°C for 30 s, and 72°C for 30 s, followed by a final extension at 72°C for 5 min. The PCR products (DNA fragments of 450–550 bp in length) were purified by 2% agarose gel electrophoresis and the GeneJET Gel Extraction Kit (Thermo Fisher Scientific, United States) before the construction of sequencing libraries. All libraries (*n* = 123) were subjected to 2 × 250 paired-end sequencing on an Illumina NovaSeq 6000 platform (Novogene Co., Ltd., Beijing, China). After trimming adaptor or barcode sequences, we obtained raw tags by merging overlapping paired-end reads with FLASH (Magoč and Salzberg, 2011). We applied QIIME (Caporaso et al., 2010) (v1.7.0) to filter raw tags with the following operations: (1) Tag truncation: cut off first low-quality base site where the number of continuous low-quality bases (Phred quality score <20) to reach the set length (default value = 3); (2) Length filtering: further remove the tags in which continuous high quality (quality score ≥ 20) base length was less than 75% of the tag length. We further compared the tags with the Gold database^1^ using the UCHIME algorithm (Edgar et al., 2011) in Usearch (v11) to identify and remove chimera sequences. We then imported tags into QIIME2 (2018.6) (Bolyen et al., 2019) and conducted bioinformatics analyses.

After denoise using the plugin DADA2 (Callahan et al., 2016) in QIIME2, the high-quality tags were clustered into bacterial features that are synonymous to sub-operational taxonomic units (sub-OTUs) (hereafter referred to as OTUs for simplicity), at a 97% sequence similarity threshold. Before conducting downstream analyses (e.g., alpha and beta diversity analyses), we discarded the OTUs with less than 10 supported tags and the OTUs that were only detected in one sample. To determine whether the sampling effort had sufficient sequence coverage to accurately describe the bacterial composition of each group, rarefaction analysis for each sampling group was conducted. To minimize the effect of sequencing depth on the estimation of alpha and beta diversities, sequences were normalized to the depth of the smallest sample. The alpha diversity (i.e., the Shannon and observed OTUs indexes) was calculated and compared among sampling groups using non-parametric tests (i.e., Kruskal–Wallis tests and Wilcoxon rank-sum tests) and the ANOVA analysis. To visualize the relationships between the samples, we performed principal coordinates analysis (PCoA) using the unweighted UniFrac and Jaccard distances. The analysis of similarity (ANOSIM) was also conducted with the Benjamini-Hochberg correction for multiple tests (*q*-value) in QIIME2. The most abundant sequence was picked for each OTU and was subjected to taxonomy annotation using the SILVA reference database (Quast et al., 2012) (v132). To identify the specific microbial taxa associated with different age groups, we conducted a comparison of the fecal microbiota using the linear discriminant analysis (LDA) effect size (LEfSe) with default parameters (Segata et al., 2011) (LDA score > 3 and *P* < 0.05), which would allow the discovery of biomarkers. We also used SourceTracker (Knights et al., 2011) with default parameters (v1.0) to explore the contributions of the maternal microbiota in different body sites to the fecal microbiota in goat kids after birth. The statistical analyses were conducted in R (R Development Core Team, 2009) (v 3.6.1), unless otherwise stated.

## Results

### Diversity of the Fecal Microbiota of Goat Kids Showed Stage-Associated Changes

The quality control of raw read pairs of all 123 samples from the goat kids (*n* = 82) and mother goats (the kids’ mothers) (*n* = 41) yielded 5,329,082 high-quality tags with an average of 43,326 tags per sample (Supplementary Table S1). Accordingly, a total of 8694 OTUs were obtained from all samples using a 97% sequence similarity threshold.

**FIGURE 1:**
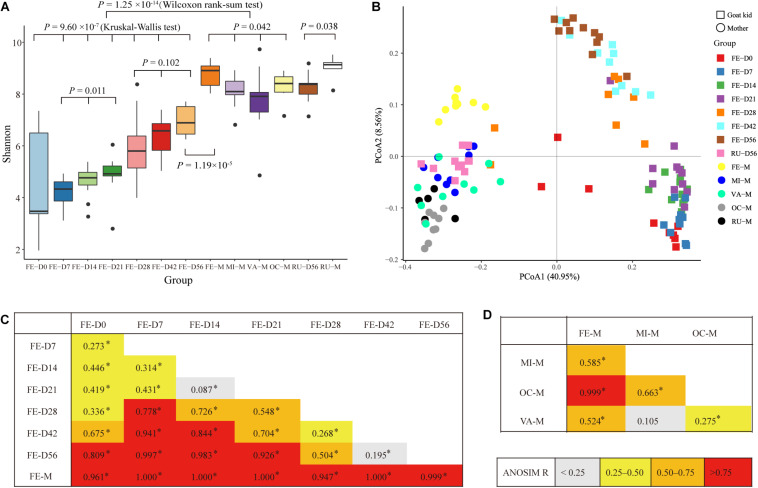
Alpha and beta diversities in all the microbial samples. **(A)** Boxplot for the Shannon diversity index in each microbial sampling group. The fecal microbiota of goat kids was sampled on days 0 (FE-D0), 7 (FE-D7), 14 (FE-D14), 21 (FE-D21), 28 (FE-D28), 42 (FE-D42), and 56 (FE-D56). The microbiota in the four body sites of mothers included oral cavity (OR-M), breast milk (MI-M), feces (FE-M), and vagina (VA-M). The rumen microbial samples were obtained from 56-day-old goat kids (RU-56) and mothers (RU-M). **(B)** Principal coordinates analysis of all 123 microbial samples using the unweighted UniFrac distance. **(C)** Analysis of similarity (ANOSIM) of the bacterial community structure among the feces of goat kids in different age groups and their mothers. High ANOSIM *R* values indicate well-separated groups. The statistical significance (*q*-value < 0.05) is indicated by ^∗^. **(D)** The ANOSIM of the bacterial community structure among four body sites of mothers.

Rarefaction curves for each sampling group using the Shannon index and the observed OTUs approached the saturation plateau when the number of tags >10,000 (Supplementary Figure S1), which suggested that the sampling had sufficient sequence coverage to accurately describe the bacterial composition of each group. Based on the Shannon index, the bacterial diversity in the feces of goat kids was lower than that in the four body sites (i.e., breast milk, feces, vagina, and oral cavity) of mothers (*P* = 1.25 × 10^–14^, Wilcoxon rank-sum test) (Figure 1A). Despite high variability in the Shannon index (2.01–7.40) on day 0, the bacterial community diversity in the feces of goat kids significantly increased with age (*P* = 9.61 × 10^–7^, Kruskal–Wallis test). We observed a slight and significant increase of the Shannon index (*P* = 0.011, Kruskal–Wallis test) in the fecal microbiota of 7- (median = 4.38) to 21-day-old (median = 4.97) goat kids. Although the alpha diversity still increased with age, there were no significant differences in the Shannon index (median of 5.85–6.94) among the feces of 28- to 56-day-old goat kids (*P* = 0.102, Kruskal–Wallis test), suggesting a more complex but similar bacterial community over time. We also found a significant difference in the Shannon index of the fecal microbiota between 56-day-old goat kids and mothers (*P* = 1.19 × 10^–5^, Wilcoxon rank-sum test). The similar richness results for all 123 samples were also indicated using the observed OTUs (Supplementary Figure S2). In addition, we applied the ANOVA analysis to compare the Shannon index and the observed OTUs among all 13 sampling groups (Supplementary Table S2), which generated the consistent results as described above.

According to the PCoA (variance explained = 40.95 and 8.56% for the first and second coordinates, respectively) using the unweighted UniFrac and Jaccard distances (Figure 1B and Supplementary Figure S3), all 123 microbial samples can be classified into four groups: the fecal samples of goat kids from birth to 21-day-old, the fecal samples of 28- to 56-day-old goat kids, the fecal samples of mothers, and the remaining microbial samples (i.e., the rumen microbiota of goat kids, and the microbiota of the breast milk, vagina, oral cavity, and rumen of mothers). Interestingly, most of the fecal microbiota samples of goat kids in different age groups were sequentially distributed along the second co-ordinate (i.e., *y*-axis).

The ANOSIM analysis using the unweighted UniFrac distance between each two age groups further showed that there were moderate differences in the bacterial community compositions among the fecal samples of goat kids on day 0 to 21 (*q*-value < 0.05, ANOSIM *R* = 0.087–0.446) (Figure 1C), which was also true among the feces of 28- to 56-day-old goat kids (*q*-value < 0.05, ANOSIM *R* = 0.195–0.504). The fecal microbiota composition of mothers showed a large difference (*q*-value < 0.05, ANOSIM *R* ≥ 0.947) with that of goat kids in each of all seven age groups (Figure 1C). Similar ANOSIM results for these samples were also generated using the Jaccard distance (Supplementary Table S3).

### The Fecal Bacterial Community of Goat Kids Was Dominated by Firmicutes, Bacteroidetes, and Proteobacteria

**FIGURE 2:**
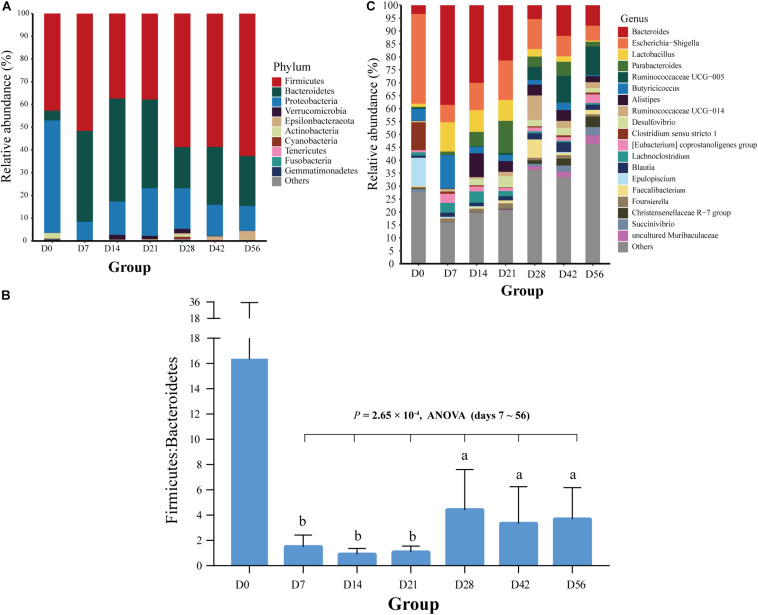
Bacterial community composition at different taxonomic levels in the feces of goat kids in all the seven age groups. **(A)** The bacterial community composition at the phylum level in feces of goat kids in the seven age groups. The top 10 bacterial phyla are represented separately with different colors, whereas the “Others” proportion represented other known phyla with low abundances and unassigned taxa. **(B)** Longitudinal changes of the Firmicutes/Bacteroidetes ratio in feces of goat kids across seven age groups. Significant differences among groups were analyzed by one-way ANOVA and Duncan’s tests. **(C)** The bacterial community composition at the genus level in feces of goat kids in seven age groups. The “Others” proportion represented other known genera with <1% average relative abundance across all samples and unassigned taxa. Different letters above the whiskers of bars indicate significantly different.

In total, 21 bacterial phyla were found in all fecal samples (*n* = 71) of goat kids from birth to 56-day-old (Supplementary Table S4). The predominant phyla were Firmicutes (49.91%), Bacteroidetes (27.73%), and Proteobacteria (19.37%), which accounted for >97% of total sequences regardless of age group, but their relative abundance varied considerably among age groups (Figure 2A). For example, the relative abundance of Proteobacteria was as high as 49.44% on day 0 and decreased sharply to 8.12% on day 7, but then slightly rose afterward. As shown in Figure 2B, high variability in the ratio of Firmicutes to Bacteroidetes was present in the feces of goat newborns, and the average ratio of Firmicutes to Bacteroidetes in the feces of kids declined rapidly from birth (16.33) to day 7 (1.51). And then, the ratio overall significantly increased from days 7, 14, and 21 to days 28, 42, and 56 (*P* = 2.65 × 10^–4^, ANOVA).

We discovered a total of 463 known bacterial genera in the feces of goat kids (Supplementary Table S4). In addition to *Bacteroides* with the highest overall average proportion (16.9%), other dominant genera belonging to Bacteroidetes consisted of *Parabacteroides* (4.4%) and *Alistipes* (3.46%) (Figure 2C). In Firmicutes, the dominant genera mainly included *Lactobacillus* (4.95%), *Ruminococcaceae UCG-005* (4.01%), *Butyricicoccus* (3.93%), *Ruminococcaceae UCG-014* (2.53%), and *Clostridium sensu stricto 1* (2.04%). Furthermore, *Escherichia-Shigella* and *Desulfovibrio*, two members of Proteobacteria, showed a high proportion of 13.27 and 2.05%, respectively.

### LEfSe Analysis Revealed Many Stage-Specific Bacterial Biomarkers

**FIGURE 3:**
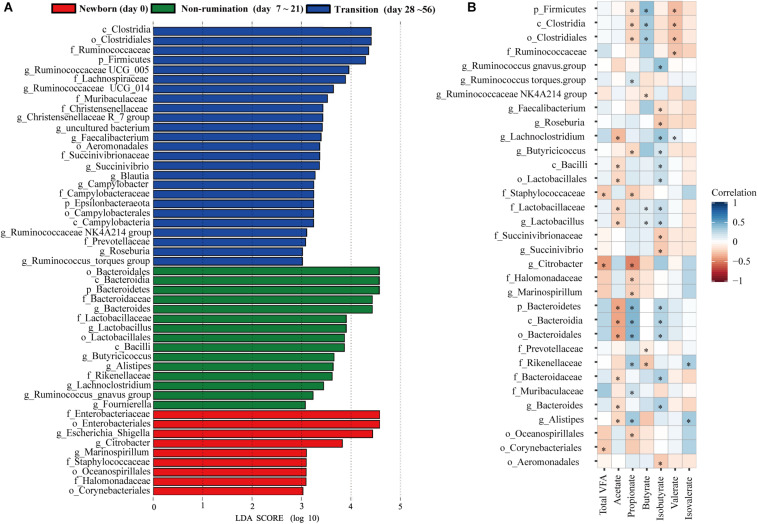
The identified biomarkers in the feces of goat kids at three growth stages and their correlations with VFAs. **(A)** The identified biomarkers in fecal samples of goat kids at three growth stages using LEfSe analysis (LDA score >3 and *P* < 0.05). **(B)** Spearman correlations between stage-associated biomarkers and VFAs in fecal samples of goat kids in seven age groups. The blue color represents a positive correlation between the biomarkers and VFAs and the red color represents a negative correlation. The statistical significance (*P* < 0.05) is denoted by ^∗^.

Based on the above analyses of diversity, similarity, and composition of the fecal microbiota, we classified the colonization of the fecal microbiota at seven time points into three different temporal stages: newborn (day 0), non-rumination (days 7–21), and transition stages (days 28–56). We identified a total of 49 unique bacterial biomarkers (Figure 3A) in the feces of goat kids among the three temporal stages using the LEfSe analysis (LDA score > 3 and *P* < 0.05), which were driven by the effects of both diet and age. Nine bacterial taxa, including *Staphylococcaceae*, *Enterobacteriaceae*, and its two members (i.e., *Escherichia-Shigella* and *Citrobacter*), were detected as biomarkers in feces of newborn goat kids (day 0). Eight and seven out of 15 biomarkers identified at the non-rumination stage (days 7–21) belonged to Firmicutes (e.g., *Lactobacillaceae*, *Lactobacillus*, and *Butyricicoccus*) and Bacteroidetes (e.g., *Bacteroidaceae*, *Bacteroides*, and *Alistipes*), respectively. In addition to Firmicutes and its 13 members (e.g., *Ruminococcaceae*, *Lachnospiraceae*, *Ruminococcaceae UCG_014*, *Ruminococcaceae UCG_005*, and *Faecalibacterium*), the biomarkers at the transition stage (days 28–56) included *Prevotellaceae* and *Muribaculaceae* belonging to Bacteroidetes, and *Succinivibrionaceae* and *Succinivibrio* belonging to Proteobacteria.

To determine the maturity levels of the fecal microbiota in 56-day-old goat kids, we also identified the bacterial biomarkers in the fecal microbiota between 56-day-old goat kids and mothers. Interestingly, 24 bacterial biomarkers, which specifically included Proteobacteria, Epsilonbacteraeota, and their members, were present in the fecal microbiota of 56-day-old goat kids (LDA score > 3 and *P* < 0.05), whereas 27 biomarkers, which specifically contained three phyla Fibrobacteres, Spirochaetes, and Verrucomicrobia, were detected in the feces of mothers (Supplementary Table S5).

### Temporal Profiles of VFAs and Their Correlations With Biomarkers in the Feces of Goat Kids

There were significant changes in proportions of all individual VFAs and concentration of total VFAs among the fecal samples of goat kids at seven different time points (*P* < 0.05, one-way ANOVA), except for isovalerate (Table 1). When compared to day 0, the concentration of total VFA and the relative concentration of propionate increased significantly on days 7 and 14 (*P* < 0.01, ANOVA), respectively, and then tended to be stable with age. However, the proportion of acetate and the ratio of acetate to propionate dropped dramatically from days 0 to 7 (*P* < 0.01, ANOVA), but did not significantly change among subsequent age groups. The highest relative concentration of butyrate was observed in the feces on day 7.

**TABLE 1:** Concentrations of VFAs in the feces of the goat kids among age groups.

VFAs	D0	D7	D14	D21	D28	D42	D56	*P*-value
Total VFA (mM)	37.49^b^	133.45^a^	145.64^a^	127.49^a^	152.63^a^	131.20^a^	142.23^a^	<0.01
Acetate (%)	84.95^a^	75.53^b^	74.49^b^	75.30^b^	74.35^b^	77.69^b^	78.23^b^	<0.01
Propionate (%)	2.22^c^	6.88^b^	10.74^a^	11.37^a^	9.28^ab^	10.47^a^	9.27^ab^	<0.01
Butyrate (%)	3.80^c^	9.52^a^	6.95^abc^	5.57^bc^	7.86^ab^	4.63^bc^	7.38^ab^	0.015
Isobutyrate (%)	3.07^b^	5.36^a^	3.52^b^	2.88^b^	3.69^b^	3.20^b^	2.19^b^	<0.01
Valerate (%)	0.85^abc^	0.37^c^	0.95^abc^	1.60^a^	1.26^ab^	0.58^bc^	0.35^c^	<0.01
Isovalerate (%)	5.11	2.34	3.35	3.28	3.57	3.44	2.58	0.074
Acetate: propionate	39.94^a^	11.86^b^	7.74^bc^	6.97^c^	8.90^bc^	7.98^bc^	9.14^bc^	<0.01

*Values with different letter superscripts within a row indicate a significant difference (*P* < 0.05, one-way ANOVA and Duncan’s tests).*

Among the 49 bacterial biomarkers identified above, 33 biomarkers showed significant Spearman correlations (*P* < 0.05) with individual VFAs or total VFA in the feces of goat kids (*n* = 71) (Figure 3B). The 11 significant acetate-associated biomarkers included Bacteroidetes, *Bacteroidaceae*, *Lactobacillaceae*, *Bacteroides*, and *Lactobacillus* (ρ < −0.17). The propionate was positively associated with seven biomarkers, including Bacteroidetes and its members (e.g., *Rikenellaceae*, *Muribaculaceae*, and *Alistipes*) (ρ > 0.17), whereas there were significant negative correlations (ρ < −0.16) between propionate and nine biomarkers (e.g., Firmicutes, *Citrobacter*, and *Marinospirillum*). Significant positive correlations were present between butyrate and the Firmicutes and its members (e.g., *Clostridia*, *Lactobacillaceae*, and *Lactobacillus*) (ρ > 0.12), and we found negative relationships with *Prevotellaceae* and *Rikenellaceae* (ρ < −0.08). Furthermore, 17 biomarkers were significantly related to isobutyrate (positive correlation: 12 biomarkers; negative correlation: 5 biomarkers).

### The Vaginal Microbiota of Mothers Was the Principal Source of the Fecal Microbiota of Goat Kids at Early Ages

**FIGURE 4:**
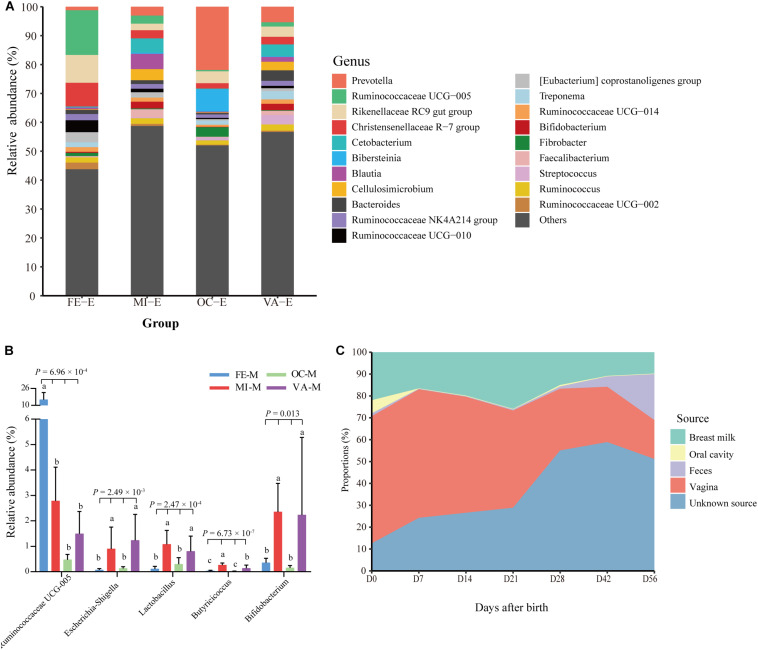
Proportion of each fecal microbiota sample in goat kids estimated to originate from different maternal body sites. **(A)** The bacterial community composition at the genus level in the four body sites of mothers. The “Others” proportion represented other remaining genera with <1% average relative abundance across all samples and unassigned taxa. **(B)** The differential proportion analysis of five bacterial genera that were highly abundant in the feces of goat kids among the four body sites of mothers. Significant differences among groups were analyzed by one-way ANOVA and Duncan’s tests. **(C)** Dynamic contributions of different microbial sources to gut microbiota in goat kids from birth to day 56 using SourceTracker. Different letters above the whiskers of bars indicate significantly different.

In this study, the microbiota in four body sites (i.e., the breast milk, feces, vagina, and oral cavity) of goat kids’ mothers was regarded as potential sources of the fecal microbiota in goat kids. Overall, there were significant differences in the Shannon diversity index (*P* = 0.042, Kruskal–Wallis test) and similarity (e.g., ANOSIM R = 0.999 between the fecal and the oral microbiota) of the microbiota among these four body sites (Figures 1A,D). For example, the dominant phyla Firmicutes was more enriched in feces (65.53%) relative to the other three body sites (*P* = 6.57 × 10^–8^, ANOVA), whereas Bacteroidetes was most abundant (42.61%), followed by Proteobacteria (24.06%), in the oral cavity (Supplementary Table S6). The proportion of Actinobacteria in the vagina (10.65%) and breast milk (8.82%) was significantly higher than that in the other two body sites (*P* < 0.01, ANOVA). At the genus level, *Prevotella* showed the highest proportion in the vaginal (5.36%) and oral (21.87%) microbiota, whereas the most abundant genera were *Ruminococcaceae UCG-005* (15.46%) and *Blautia* (5.31%) in the fecal and breast milk microbiota of mothers, respectively (Figure 4A). As a well-known beneficial bacterial genus, the relative abundance of *Bifidobacterium* in the breast milk (2.35%) and vagina (2.23%) was significantly higher (*P* < 0.05, ANOVA) than that in the other two body sites of mothers. Interestingly, four (i.e., *Escherichia-Shigella*, *Lactobacillus*, *Ruminococcaceae UCG-005*, and *Butyricicoccus*) highly abundant bacterial genera in feces of goat kids showed significantly different proportions (*P* < 0.05, ANOVA) from the microbiota in the four body sites of mothers (Figure 4B).

The results obtained by using SourceTracker showed that, on average, 40.81 and 17.12% of the fecal microbiota were from the vagina and breast milk of mothers (Figure 4C), respectively, suggesting that both body sites were the main sources especially in early age groups (days 0–21). However, the contribution of the microbiota in the breast milk and vagina of mothers to the fecal microbiota of kids generally declined with the age of kids (breast milk: 21.95 to 9.78%, vagina: 58.15 to 17.78%). In contrast, maternal fecal microbiota of mothers accounted for a growing proportion (1.32 to 21.02%) from day 0 to day 56. The contribution of the microbiota from unknown sources rose gradually from day 0 to day 21 but showed a rapid upward trend after day 28, which reflected the contributions of environmental sources, especially the roughage.

### The Ruminal Bacterial Compositions and VFA Concentrations in 56-Day-Old Goat Kids Were Similar to Those in Mothers

**FIGURE 5:**
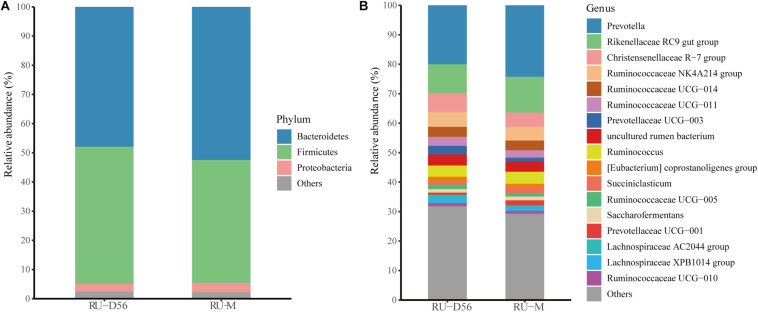
Bacterial community compositions at different taxonomic levels in the rumen fluids of the 56-day-old goat kids and mothers. **(A)** The bacterial community composition at the phylum level in the rumen fluids of the 56-day-old goat kids and mothers. The “Others” proportion represented other remaining phyla with low abundances. **(B)** The bacterial community composition at the genus level in the rumen fluids of the 56-day-old goat kids and mothers. The “Others” proportion represented other remaining genera with <1% average relative abundance across all samples.

The Shannon diversity index of the rumen microbiota in 56-day-old goat kids (median = 8.422) was moderately less (*P* = 0.038, Wilcoxon rank-sum test) than that in their mothers (median = 9.046) (Figure 1A), and there was no significant difference (*P* = 0.115) in richness (i.e., the observed OTUs) between them (Supplementary Figure S2). The ANOSIM analysis showed a significant but moderate difference in the rumen microbiota between 56-day-old goat kids and mothers (ANOSIM *R* = 0.532, *q*-value < 0.05) (Supplementary Table S3). However, the relative abundances of Bacteroidetes (47.9 and 52.44%), Firmicutes (47.03 and 42.14%), and Proteobacteria (2.51 and 3.15%) that were the dominant bacterial phyla did not show significant differences in the rumen between 56-day-old goat kids and mothers (*P* > 0.05, Wilcoxon rank-sum test) (Figure 5A). Among the 401 genera (Supplementary Table S7), *Prevotella* showed the highest relative abundances (20.00 and 24.21%) in the rumen of 56-day-old goat kids and mothers, followed by *Rikenellaceae RC9 gut group* (9.73 and 12.19%), *Christensenellaceae R-7 group* (6.49 and 4.83%), and *Ruminococcaceae NK4A214 group* (5.01 and 4.06%) (Figure 5B). LEfSe analysis (LDA score > 3 and *P* < 0.05) only revealed four bacterial taxa in the comparison of the rumen microbiota between both groups (Supplementary Table S8). There were no significant differences (*P* > 0.05) in the concentration of total VFA and proportions of individual VFAs in the rumen between 56-day-old goat kids and mothers, except for butyrate and valerate (Table 2).

**TABLE 2:** Concentrations of VFAs in the rumen of the 56-day-old goat kids and their mothers.

VFAs	RU-D56	RU-M	*P*-value
Total VFA (mM)	63.98	77.44	0.33
Acetate (%)	72.77	71.39	0.26
Propionate (%)	15.88	14.96	0.32
Butyrate (%)	8.20^a^	4.19^b^	<0.01
Isobutyrate (%)	2.55	2.33	0.15
Valerate (%)	3.04^a^	1.99^b^	0.02
Isovalerate (%)	1.56	1.11	0.25
Acetate: propionate	4.80	4.82	0.97

*Values with different letter superscripts within a row indicate a significant difference.*

## Discussion

In this study, we mainly reported how the gut microbiota acquires and develops in goat kids during the first 8 weeks (i.e., from birth to the rumination phase) of life, and in particular the role of different maternal sources in this process, by analyzing 123 microbial samples from 11 healthy goat kids and their mothers. One finding of this study is that the colonization of the gut microbiota in Chengdu Brown goat kids showed stage-associated changes. Due to the development of the four stomach compartments after birth, the digestive physiological process of ruminants should shift from non-rumination to rumination. According to the temporal characteristics of four stomachs especially with the rumen, the development of the gastrointestinal system in sheep can be divided into three temporal stages: non-rumination (0–21 days), transition (21–56 days), and rumination stages (from 56 days onward) (Wardrop and Coombe, 1960; Lane et al., 2002). However, direct evidence from goats was limited, and recent longitudinal analyses showed that the rumen (Jiao et al., 2015b; Zhang et al., 2019) and ileal (Jiao et al., 2016) microbiota in goat kids underwent dramatic changes in three stages during the first 2 months after birth. Furthermore, other studies showed that the first 2 days of life were the initial phase of the gut microbiota establishment in ruminants, according to the analyses of the ruminal microbiota and VFA concentrations in calves (Rey et al., 2014) and goat kids (Abecia et al., 2014). Based on the differences in alpha and beta diversities, as well as the composition of the dominant bacteria across the age groups, we classified the development of the gut microbiota in Chengdu Brown goat kids during the first 56 days of age into three temporal stages (i.e., day 0, days 7–21, and days 28–56), which likely resulted from the cumulative effects of age and diet changes. However, we cannot discriminate between age and diet effects because of the progressive changes of dietary intake with age, although the intake of solid feed for the goat kids generally started at ∼20 days in our study.

The substantial differences in beta diversity and many identified bacterial biomarkers suggested that the fecal microbiota of 56-day-old goat kids is far less mature compared to that in their mothers. Yet, the relative abundance of dominant phyla (i.e., Firmicutes, Bacteroidetes, and Proteobacteria) in the rumen microbiota of 56-day-old kids was comparable to that in their mothers and only a few bacterial biomarkers were detected, although the alpha and beta diversities showed significant differences. Furthermore, the concentration of the ruminal total VFA, acetate, propionate also showed substantial similarities between 56-day-old goats and mothers. Therefore, we suggest that the core rumen microbiota of goat kids at 56 days is close to that in adult goats and is thus relatively mature.

### The Gut Microbial Composition in the Feces of Goat Kids in Early Life

Like the findings in feces of humans (Zhang et al., 2009; Fallani et al., 2011; Koenig et al., 2011; Faith et al., 2013) and animals [e.g., cattle (Durso et al., 2010; Alipour et al., 2018), sheep (Bi et al., 2019), pigs (Looft et al., 2014), and monkeys (Yasuda et al., 2015)], the phyla Firmicutes, Bacteroidetes, and Proteobacteria were predominant in the fecal and rumen microbiota of goat kids and their mothers. The highest abundance of Proteobacteria and high variability in alpha diversity were present in the fecal microbiota of goat newborns on day 0, which is also true in hindguts of other goat breeds (Jiao et al., 2016; Li et al., 2019) and the rumen of calves (Rey et al., 2014). The initial colonization of facultative anaerobic Proteobacteria scavenges oxygen and is beneficial to the subsequent rapid growth of obligate anaerobes such as Firmicutes and Bacteroidetes (Shin et al., 2015). Similarly, the neonatal intestinal microbiota is initially dominated by Proteobacteria that is soon replaced by Firmicutes and Bacteroidetes in human infants (Eckburg et al., 2005; Qin et al., 2010; Fallani et al., 2011; Koenig et al., 2011). We also found stage-associated differences in the ratio of the fecal Firmicutes to Bacteroidetes, which can be attributed to the combined effects of age and diet changes.

### Stage-Specific Bacterial Biomarkers in the Feces of Goat Kids in Early Life

We revealed dramatic shifts of the fecal bacterial composition in goat kids from birth to day 56 using LEfSe (Segata et al., 2011), which provided a better understanding of the colonization of the hindgut microbiota in ruminants in early life. The distinct signature bacteria in feces of goat newborns included *Enterobacteriaceae* and its two members (i.e., *Escherichia-Shigella* and *Citrobacter*) that are also abundant in infant fecal microbiota (Vallés et al., 2014; Milani et al., 2017; Lackey et al., 2019). As a class of facultative anaerobic bacteria, *Escherichia-Shigella* is present in the gut microbiota of infants (Bäckhed et al., 2015) and infant animals (Liu et al., 2019). Although they are generally regarded as potential pathogens, some members of *Escherichia* can help anaerobic bacteria create an anaerobic environment by scavenging oxygen (Jones et al., 2007, 2011). In turn, anaerobic bacteria can degrade complex polysaccharides into the disaccharides and monosaccharides that are needed for the growth of Escherichia (Jones et al., 2007, 2011), which support a high abundance of *Escherichia-Shigella* in feces of goat newborns.

Here, the bacterial biomarkers for the goat kids during the non-rumination stage (i.e., days 7–21) were mainly Bacteroidetes and its members (e.g., *Bacteroidaceae*, *Bacteroides*, and *Alistipes*) and several members in Firmicutes (e.g., *Lactobacillaceae*, *Lactobacillus*, and *Butyricicoccus*), which can be attributed to the breast milk-dominated feeding that is rich in lactose, protein, and fat. These results are similar to the findings for the fecal microbiota in infants (Vallés et al., 2014; Milani et al., 2017) and calves (Klein-Jöbstl et al., 2014), as well as the ileal microbiota (Malmuthuge et al., 2014), in preweaning calves. In humans, it is generally recognized that the *Bacteroides*-dominated enterotype is driven by a high-fat and high-protein diet which includes animal fat and milk (Arumugam et al., 2011; Wu et al., 2011). The production of a novel donkey milk fermented beverage demonstrated the ability of *Lactobacillaceae* to utilize carbohydrates and other nutrients in milk (Turchi et al., 2017). As a relatively new genus in the phylum Bacteroidetes, the knowledge of *Alistipes* is limited to date (Polansky et al., 2016), and a previous study showed that *Alistipes* was resistant to bile (Rautio et al., 2003). Although it was present with a relatively high proportion (>0.44%) in feces of goat kids throughout the first 56 days of life, *Lactobacillus* was particularly abundant at the non-rumination stage and was thus identified as a biomarker. Previous studies have demonstrated that *Lactobacillus* could enhance the protection of the human gut from different intestinal infections (Tamburini et al., 2016) and is associated with the production of beneficial metabolites (Arboleya et al., 2015).

It was not surprising that several cellulolytic bacteria (e.g., *Lachnospiraceae* and *Ruminococcaceae*) and *Prevotellaceae* were identified as biomarkers at the transition stage (i.e., days 28–56), because a large amount of roughage intake for the goat kids in this study began at ∼20 days. A previous study has revealed that many genes in the genomes of *Lachnospiraceae* and *Ruminococcaceae* encode carbohydrate-active enzymes, and thus both families serve as plant degraders (Biddle et al., 2013). The higher abundance of *Ruminococcaceae UCG_005* compared to captive individuals was observed in feces of wild forest musk deer, which was explained that wild forest musk deer mainly consumed wild high-fiber plant leaves in their diets (Li et al., 2017). Furthermore, an increased abundance of *Prevotella* in human feces is positively correlated with high fiber intake (De Filippo et al., 2010; Wu et al., 2011; Kovatchevadatchary et al., 2015). Therefore, we concluded that a diet rich in high fiber and other plant-derived compounds mainly drove the rapid colonization of these biomarkers in the intestine of goat kids during the transition stage.

### Correlations Between Fecal Microbiota and VFAs in Goat Kids

VFAs, mainly including acetate, butyrate, and propionate are microbially produced metabolites from unabsorbed/undigested diet components in the intestine and in particular the colon of the host (Ríos-Covián et al., 2016). It is well known that most enteric bacteria are capable of producing acetate (Koh et al., 2016). However, we found that Bacteroidetes and several of its members (e.g., *Bacteroidia*) showed significant negative correlations with the proportion of acetate in feces of goat kids, which was consistent with previous findings in obese children (Riva et al., 2017) and rats fed with high-fat diet (Lin et al., 2016). The production of propionate in the colon is mainly contributed by Bacteroidetes via three pathways, for which the succinate pathway is the major one (Flint et al., 2012; Reichardt et al., 2014). Thus, the fecal relative propionate concentration was linked to the abundance of Bacteroidetes in humans (Salonen et al., 2014), and two genera (i.e., *Alistipes* and *Bacteroides*) in Bacteroidetes were positively related with the production of propionate in chicken cecum (Polansky et al., 2016). Similarly, we observed that propionate was positively correlated with Bacteroidetes, whereas it showed a negative association with Firmicutes. Interestingly, fructo-oligosaccharide supplementation results in increasing levels of Bacteroidetes and a reduction of Firmicutes, which enhances the proportion of propionate in the portal vein (De Vadder et al., 2014) in mice.

Butyrate is the preferred energy source for colonocytes and most butyrate-producing bacteria in the human colon belong to the Firmicutes phylum, in particular with Clostridium bacteria (Louis and Flint, 2009; Den Abbeele et al., 2013; Vital et al., 2014). Here, we found that Firmicutes and several members (e.g., *Clostridia* and *Lactobacillaceae*) in this phylum enhanced butyrate formation in feces of goat kids as inferred using correlation analysis. Similarly, there is a positive correlation between *Lactobacillaceae* and overall fecal butyrate concentration in dogs (Gagne et al., 2013). *Lactobacillus rhamnosus* GG-supplemented formula expanded butyrate-producing bacteria in the infant gut, which leads to an increase of the fecal butyrate level (Canani et al., 2016). Fecal isobutyrate and other branched-chain fatty acids are from protein degradation and account for a small proportion of total VFAs (Koh et al., 2016). Isobutyrate supplementation can improve rumen microbiota, enzyme activities, and methane emissions in steers (Wang et al., 2015). Consistent with previous reports in mice (Ilhan et al., 2017) and chicken (Wang et al., 2017), we found that the fecal isobutyrate level was significantly related to the abundance of several bacterial taxa (e.g., Bacteroidetes, *Bacteroides*, and *Bacilli*) in goat kids.

*Staphylococcaceae*, *Corynebacteriales*, and *Citrobacter* were negatively correlated with the abundance of total VFAs in the feces of goat kids, suggesting that these bacteria may inhibit the production of VFAs in the gut. Previous studies have reported that some species in *Corynebacteriales* (Torkko et al., 2002; Tauch et al., 2005) and *Staphylococcaceae* (David and Daum, 2010; Ibarra et al., 2013) were pathogens of humans and animals. However, their abundance decreased rapidly with age, and thus these bacteria can be thought to be only transients in the gut of goat kids. Notably, cross-feeding interactions among different bacteria can modify causal relationships between the production of VFAs and bacteria (Ríos-Covián et al., 2016). Therefore, we cannot confirm that these significant correlations are causal.

### Acquisition of the Gut Microbiota in Goat Kids

A key finding of this study is that the vaginal microbiota of mothers was the principal source of the fecal microbiota of goat kids at early growth stages. All the goat kids were born vaginally and our results are consistent with the observations in vaginally born infants (Mändar and Mikelsaar, 1996; Bezirtzoglou, 1997; Dominguez-Bello et al., 2010; Sakwinska et al., 2017). Maternal breast milk appeared to be another important source of the microbiota for goat kids, and similar results were recently reported in suckling piglets (Liu et al., 2019). Breast milk not only provides nutrients and immune-active substances for infants, but also includes several beneficial bacteria (e.g., *Lactobacillus* and *Bifidobacterium*) that may be transmitted to the intestinal tract of infants through breast-feeding (Martín et al., 2003; Solís et al., 2010; Pannaraj et al., 2017).

In this study, *Lactobacillus* was highly abundant in feces of goat kids and mothers’ milk and vagina, consistent with the findings that approximately one-quarter of infants acquire *Lactobacilli* from their mothers’ vagina at birth (Matsumiya et al., 2002). Notably, the development of the digestive tract of ruminants undergoes the transition of from non-rumination to rumination from 21 to 28 days old, due to the rapid growth of the rumen. Here, we found a large contribution (∼50%) of unknown sources to the fecal microbiota of goat kids after 28 days when the substantial solid feed intake of the goat kids was increasing, which might be attributed to the effects of the microbiota in the solid feed (corn silages and fresh grasses). However, the unknown microbial composition in the solid feed in this study hampered in-depth analyses. We also observed that the maternal fecal microbiota to some extent contributed to the fecal microbiota of goat kids since day 28, in line with the findings in humans (Mändar and Mikelsaar, 1996; Bezirtzoglou, 1997). For example, the analysis of the microbiota from multiple body sites in 25 mother-infant pairs showed that the contribution of maternal intestinal microbiota to the fecal microbiota of infants increased gradually with age (Ferretti et al., 2018).

Taken together, we found that the gut bacterial community in goat kids after birth was mainly acquired from the maternal vagina and breast milk, and its establishment occurred in three distinct phases driven by diet changes. Although they are needed to be validated in a larger number of animals and in other locations to avoid possible bias, our results provided a better understanding of the roles of lower gastrointestinal tract microbiomes in young ruminants.

## Data Availability Statement

The datasets presented in this study can be found in online repositories. The names of the repository/repositories and accession number(s) can be found below: https://www.ncbi.nlm.nih.gov/, PRJNA646846.

## Ethics Statement

The animal study was reviewed and approved by the Institutional Animal Care and Use Committee of the College of Animal Science and Technology, Sichuan Agricultural University (No. DKYB20081003).

## Author Contributions

HZ managed the grants and supervised the laboratory work. JG, HZ, and LL conceived and designed this study. PL and LZ managed the sampled goats and collected the samples. JG, PL, KZ, and XW extracted DNA and conducted the measurement of VFAs and bioinformatics analyses of 16S rRNA sequencing data. JG, PL, and KZ drafted the manuscript. All authors read and approved the final manuscript.

## Conflict of Interest

The authors declare that the research was conducted in the absence of any commercial or financial relationships that could be construed as a potential conflict of interest.

## Footnotes

1 http://drive5.com/uchime/
