# Supplementary material for: Distinct Stage Changes in Early-Life Colonization and Acquisition of the Gut Microbiota and Its Correlations With Volatile Fatty Acids in Goat Kids
Source: Front Microbiol. 2020 Oct 9;11:584742. doi: 10.3389/fmicb.2020.584742 (PMC7581860; doi:10.3389/fmicb.2020.584742)
Supplement: Supplementary file 1 [file Data_Sheet_1.ZIP › Supplementary_materials_0902/Supplementary Figures.pdf]

Supplementary Figures

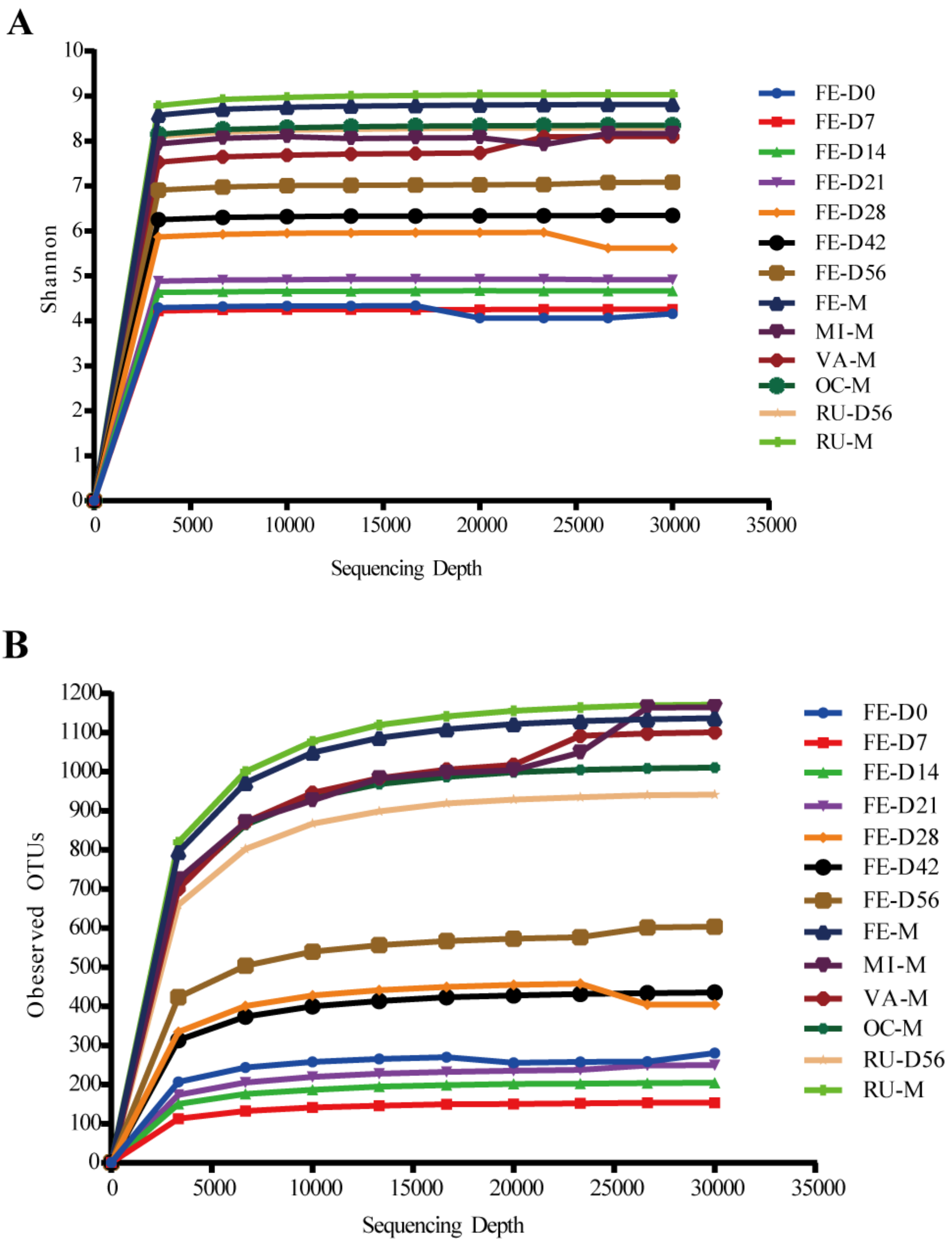

Supplementary Figure 1 Rarefaction analyses of all sampling groups.
(A) Rarefaction curves of all sampling groups based on the Shannon index (one curve per group). (B) Rarefaction curves of all sampling groups based on the observed OTUs (one curve per group).

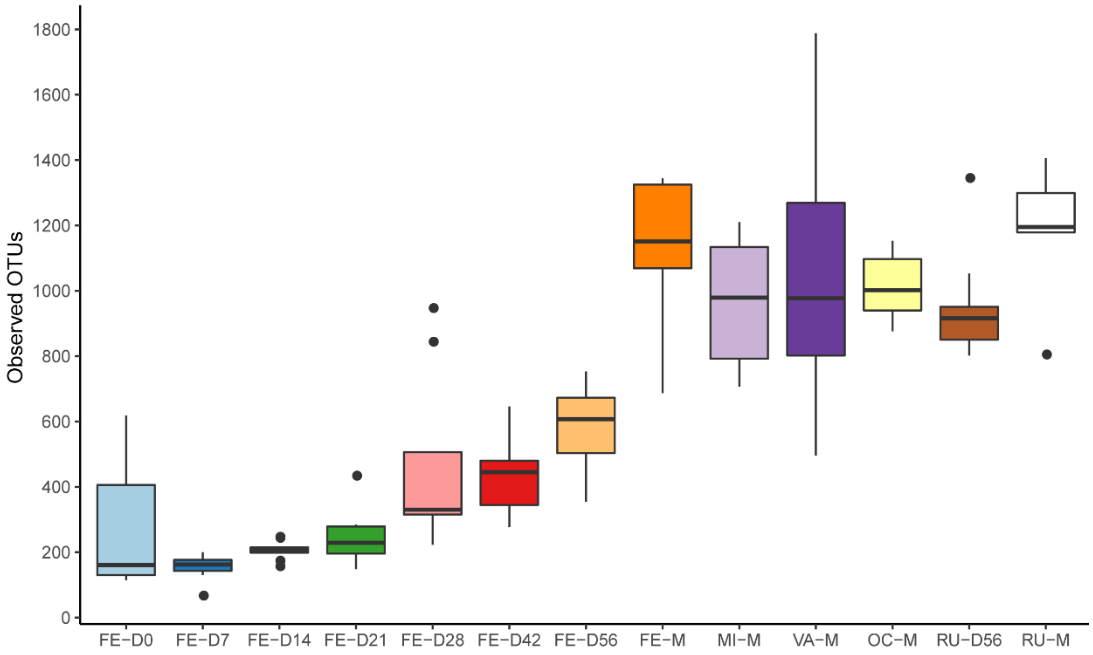

Supplementary Figure 2 Boxplot for the observed OTUs in each microbial sampling group.

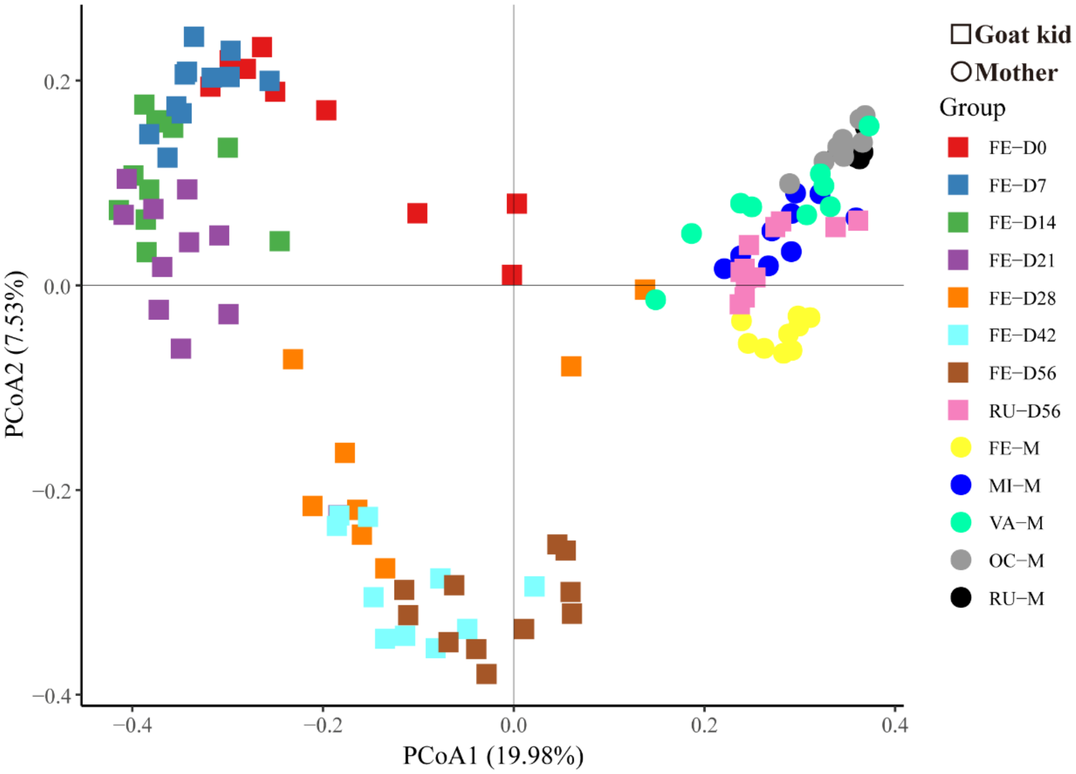

Supplementary Figure 3 Principal coordinates analysis of all 123 microbial samples using Jaccard distance.
